# Unveiling Insights into the Whole Genome Sequencing of *Mycobacterium* spp. Isolated from Siamese Fighting Fish (*Betta splendens*)

**DOI:** 10.3390/ani14192833

**Published:** 2024-10-01

**Authors:** Nguyen Dinh-Hung, Samuel Mwakisha Mwamburi, Ha Thanh Dong, Channarong Rodkhum, Watcharachai Meemetta, Nguyen Vu Linh, Hung N. Mai, Arun K. Dhar, Ikuo Hirono, Saengchan Senapin, Satid Chatchaiphan

**Affiliations:** 1 Aquaculture Pathology Laboratory, School of Animal & Comparative Biomedical Sciences, The University of Arizona, Tucson, AZ 85721, USA; dinhhung@arizona.edu (N.D.-H.); hungmai@arizona.edu (H.N.M.); adhar@arizona.edu (A.K.D.); 2 Kenya Marine and Fisheries Research Institute, Mombasa 80100, Kenya; mwakishasam@gmail.com; 3 Laboratory of Genome Science, Tokyo University of Marine Science and Technology, Tokyo 108-8477, Japan; hirono@kaiyodai.ac.jp; 4 Aquaculture and Aquatic Resources Management (AARM), School of Environment, Resources and Development, Asian Institute of Technology (AIT), Pathum Thani 12120, Thailand; htdong@ait.asia; 5 Center of Excellence in Fish Infectious Diseases (CE FID), Department of Veterinary Microbiology, Faculty of Veterinary Science, Chulalongkorn University, Bangkok 10330, Thailand; channarong.r@chula.ac.th; 6 Fish Health Platform, Center of Excellence for Shrimp Molecular Biology and Biotechnology (Centex Shrimp), Faculty of Science, Mahidol University, Bangkok 10400, Thailand; w.meemetta@gmail.com; 7 National Center for Genetic Engineering and Biotechnology (BIOTEC), National Science and Technology Development Agency (NSTDA), Pathum Thani 12120, Thailand; 8 Department of Animal and Aquatic Sciences, Faculty of Agriculture, Chiang Mai University, Chiang Mai 50200, Thailand; linhvu.n@cmu.ac.th; 9 Department of Aquaculture, Faculty of Fisheries, Kasetsart University, Bangkok 10900, Thailand

**Keywords:** average nucleotide identity, Betta splendens, genomic sequencing, non-tuberculous mycobacteria, whole genome

## Abstract

**Simple Summary:**

This first comprehensive genomic analysis of rapidly growing non-tuberculous mycobacteria (RGM) isolates from *Betta splendens* has led to the re-identification of certain isolates and the discovery of a potential new species, *Mycobacterium mucogenicum* subsp. *phocaicum* sp. nov. The in silico analysis is consistent with and confirms previously reported in vitro studies on virulence and antibiotic susceptibility profiles. This study emphasizes the crucial role of whole genome sequencing in the accurate identification and classification of mycobacterial species.

**Abstract:**

This study aims to genomically elucidate six isolates of rapidly growing non-tuberculous mycobacteria (RGM) derived from Siamese fighting fish (*Betta splendens*). These isolates had previously undergone phenotypic and biochemical characterization, antibiotic susceptibility testing, and in vivo virulence assessment. Initial DNA barcoding using the 16S rRNA sequence assigned these six isolates to five different species, namely *Mycobacterium chelonae* (BN1983), *M. cosmeticum* (BN1984 and N041), *M. farcinogenes* (SNSK5), *M. mucogenicum* (BN1956), and *M. senegalense* (BN1985). However, the identification relied solely on the highest percent identity of the 16S rRNA gene, raising concerns about the taxonomic ambiguity of these species. Comprehensive whole genome sequencing (WGS) and extended genomic comparisons using multilocus sequence typing (MLST), average nucleotide identity (ANI), and digital DNA–DNA hybridization (dDDH) led to the reclassification of BN1985 and SNSK5 as *M. conceptionense* while confirming BN1983 as *M. chelonae* and BN1984 and N041 as *M. cosmeticum*. Notably, the analysis of the BN1956 isolate revealed a potential new species that is proposed here as *M. mucogenicum* subsp. *phocaicum* sp. nov. Common genes encoding “mycobacterial” virulence proteins, such as PE and PPE family proteins, MCE, and YrbE proteins, were detected in all six isolates. Two species, namely *M. chelonae* and *M. cosmeticum*, appear to have horizontally acquired T6SS-II (*clpB*), catalase (*katA*), GroEL (*groel*), and capsule (*rmlb*) from distantly related environmental bacteria such as *Klebsiella* sp., *Neisseria* sp., *Clostridium* sp., and *Streptococcus* sp. This study provides the first draft genome sequence of RGM isolates currently circulating in *B. splendens* and underscores the necessity of WGS for the identification and classification of mycobacterial species.

## 1. Introduction

The genus *Mycobacterium* is characterized by the presence of mycolic acids in the cell wall, aerobic growth, and a bacillary form, with *Mycobacterium tuberculosis* serving as the type strain [1]. Currently, it comprises over 190 species, including significant human pathogens such as *M. tuberculosis* and *Mycobacterium leprae*, as well as opportunistic non-tuberculous mycobacteria (NTM) [2]. Traditionally, the genus has been classified by growth rate and further subdivided into complexes or groups [3]. The GenBank database (https://www.ncbi.nlm.nih.gov/, accessed on 19 May 2024) and the partners of the International Nucleotide Sequence Database Collaboration (http://www.insdc.org/, accessed on 19 May 2024) now contain the 16S rRNA sequences of thousands of officially recognized mycobacterial strains. It is, therefore, not surprising that taxonomic and phylogenetic studies of mycobacterial species have been based primarily on 16S rRNA for several decades [4]. The 16S rRNA gene-based taxonomic classification has been largely adequate in the separation of rapidly and slowly growing species and the grouping of most species into well-defined phylogenetic groups (complexes) [5]. However, the utility of 16S rRNA gene-based classification in differentiating closely related mycobacterial species is limited due to a very high sequence similarity among mycobacteria, making precise phylogenetic differentiation difficult [6,7]. In contrast, whole genome sequencing has been shown to be more effective than traditional genotyping in delineating species, discovering new species, and investigating specific traits [8,9,10]. Therefore, the application of whole genome analysis represents a significant step forward in mycobacterial research and provides deeper insights into the genetic diversity and complexity of the genus *Mycobacterium*.

We recently conducted a comprehensive investigation into the phenotypic and biochemical characteristics, as well as the antibiotic and disinfectant susceptibility of rapidly growing non-tuberculous mycobacteria (RGM) isolated from Siamese fighting fish (*Betta splendens*) [11]. Specifically, the study included six isolates representing five RGM species, namely *Mycobacterium chelonae* (BN1983), *M. cosmeticum* (BN1984 and N041), *M. farcinogenes* (SNSK5), *M. mucogenicum* (BN1956), and *M. senegalense* (BN1985), that were initially identified based on the 16S rRNA gene sequence. Additionally, we evaluated the virulence and pathogenicity of these isolates towards their host species by experimentally infecting fish through intraperitoneal injection. The results demonstrated the pathogenic nature of these rapidly growing NTMs to *B. splendens* and revealed the presence of multiple antibiotic-resistant patterns using in vitro assays. Notably, the *M. chelonae* (BN1983) isolate exhibited the highest virulence and resistant to multiple antibiotics. This underscores the significance of this group of RGM and provides the impetus for a comparative genomic analysis of these closely related mycobacterial species.

As a follow-up of our previous studies [11,12,13] we present the draft genomes of all six isolates derived from Siamese fighting fish (*B. splendens*), marking the first comprehensive genomic analysis of these isolates. The whole genome sequence analysis for each isolate focuses on describing genomic features; clarifying taxonomic identification using MLST, ANI, and dDDH; and profiling virulence factors and antimicrobial resistance genes. The findings identified the BN1956 isolate as a potential novel species, which we propose to name *Mycobacterium mucogenicum* subsp. *phocaicum* sp. nov. This study underscores the potential risks of misidentifying *Mycobacterium* species when relying solely on a single or limited set of target genes, highlighting the complex taxonomic landscape of these pathogens.

## 2. Materials and Methods

### 2.1. DNA Extraction and Sequencing

The bacterial samples were isolated from Siamese fighting fish, *B. splendens,* as described in our previous studies [11,14]. The total of six RGM isolates used in this study were obtained from naturally occurring fish displaying signs of either big belly syndrome (BBS) or skin nodule syndrome (SNS), as previously described by Dong et al. [14].

The isolates were cultured at 30 °C for 2 days in 5 mL sterile Middlebrook 7H9 broth (HiMedia, Mumbai, India) containing 10% oleic albumin dextrose catalase growth supplement. The bacterial cultures were then pelleted by centrifugation at 5000× *g* for 5 min, and total genomic DNA was extracted using the phenol–chloroform extraction method [15,16]. Briefly, the harvested bacterial cells were lysed using EDTA-saline, RNase A, and lysozyme. Subsequently, SDS and proteinase K were added, followed by 3 rounds of phenol:chloroform:isoamyl alcohol extraction. After purification, DNA was precipitated using sodium acetate and ethanol, washed with 70% ethanol, and resuspended in nuclease-free water. The quality and quantity of the extracted DNA were assessed using Nanodrop and Qubit instruments. Short-insert library preparation and sequencing were performed at BGI-Hong Kong (Hong Kong, China) using the DNBseq^TM^ platform.

### 2.2. Whole Genome Assembly and Annotation

The raw data were filtered to remove contaminants, adapters, and low-quality sequences using SOAPnuke v2.1.8 [17]. The following filter parameters were applied: “ -n 0.01 -l 20 -q 0.4 –adaMis 3 –outQualSys 1 –minReadLen 150”, which removed reads that matched ≥ 50.0 with the adapter sequence and mismatched, at most, 3 bases. In addition, sequence reads with a quality score < 20 and a proportion ≥40.0% of the total reads, as well as reads shorter than 150 bp and reads with N content ≥ 1.0%, were discarded.

Whole genome de novo assembly of six isolates was performed with Read Assembly and Annotation Pipeline Tool (RAPT) build rapt-45639894 using SKESA version 2.5.1 [18], the taxonomic assignment tool ANI [19], and Prokaryotic Genome Annotation Pipeline (PGAP) 2023-05-17 build6771 [20]. In addition, another round of annotation was performed with Prokka version 1.14.6 [21] for enhanced confirmation. Genome maps for the draft genomes were generated using Proksee (https://proksee.ca, accessed on 27 February 2024) [22]. Mobile genetic elements were discovered and characterized with mobileOG-db [23].

### 2.3. Analysis of the Assembled Draft Genomes

#### 2.3.1. Genome-Based Taxonomic Analysis

The assembled genomes were analyzed using rMLST [24] and the Type (Strain) Genome Server (TYGS) (https://tygs.dsmz.de, accessed on 1 January 2024), employing a 70% digital DNA–DNA hybridization (dDDH) cutoff for a whole genome-based taxonomic analysis [25,26]. Briefly, the determination of the genomes of the closest type strains was performed in two complementary ways. First, all genomes in our study were compared to genomes of type strains available in the TYGS database using the MASH algorithm and a fast approximation of intergenomic relatedness [27]. Then, ten type strains with the smallest MASH distances per genome were selected. Secondly, an additional group of ten closely related type strains was determined based on the 16S rDNA gene sequences. These were extracted from our study genomes using RNAmmer [28], and each sequence was then compared to the 16S rDNA gene sequence of each of the 20,173 type strains currently available in the TYGS database using BLASTed [29]. This was used as a proxy to find the best 50 matching type strains (according to the bitscore) for each of our study genomes; then, exact distances were calculated using the Genome BLAST Distance Phylogeny (GBDP) approach under the “coverage” algorithm and distance formula *d5* [30]. These distances were finally used to determine the ten closest type-strain genomes for each of the six genomes we sequenced. For phylogenomic inference, all pairwise comparisons between genomes were performed using GBDP, and exact intergenomic distances were inferred using the “trimming” algorithm and distance formula *d5* [30]. For each comparison, 100 distance replicates were calculated. The dDDH values and confidence intervals were calculated using the recommended settings of GGDC version 4.0 [26,30]. The average nucleotide identity (ANI) values of our study genomes compared to the respective reference genomes determined in the previous step were calculated using OrthoANIu [31] and FastANI version 1.34 [32].

#### 2.3.2. Clustering and Phylogenetic Inference

Species clustering was performed using a 70% dDDH threshold, grouping around each of the 60 type strains to differentiate species [25]. For subspecies, a stricter 79% dDDH threshold was applied to ensure more precise clustering [33]. The resulting intergenomic distances were used to construct a balanced minimum-evolution tree that was generated using FASTME version 2.1.6.1, with subtree pruning and regrafting (SPR) post processing to optimize the tree topology [34]. Branch support was calculated from 100 pseudo-bootstrap replicates to enhance the reliability of the tree. The final tree was centrally rooted to provide a clearer visualization of evolutionary relationships [35] and was displayed using the interactive PhyD3 tool for better interpretability [36].

#### 2.3.3. Plasmid Identification and Antimicrobial Resistance Profiling

Multilocus sequence typing analysis (MLST) was performed for housekeeping genes S142Z, L35, S19, L19, S12, S8, L16, and S7, together with their corresponding sequence types (STs). Plasmid sequences were identified within the draft genomes using Plasmer version 0.1 20220816 [37], which employs machine learning to analyze shared k-mers and genomic features. The identified plasmid sequences were then taxonomically classified by comparing them to all plasmids in the NCBI RefSeq database using Kraken 2 version 2.1.2 [38]. Antimicrobial resistance genes (ARGs) were detected using several complementary tools to ensure comprehensive identification across various databases and detection methods. ResFinder version 4.4.2 [39] was chosen for its accuracy in identifying acquired antimicrobial resistance genes based on curated reference sequences. abriTAMR version 3.10.42 [40], supported by AMRFinderPlus [41], was employed to enhance sensitivity in detecting ARGs by comparison against multiple databases. Additionally, ABRicate v1.0.1 (https://github.com/tseemann/abricate, accessed on 28 February 2024) was used to scan contig sequences against various ARG databases, including NCBI AMRFinderPlus [41], CARD [42], Resfinder [43], ARG-ANNOT [44], VFDB [45], PlasmidFinder [46], EcOH [47], and MEGARes 2.0 [48]. The Resistance Gene Identifier (RGI) online portal version 6.0.3 (https://card.mcmaster.ca/analyze/rgi, accessed on 28 February 2024) was also used to predict resistomes by integrating homology and SNP models, leveraging the Comprehensive Antibiotic Resistance Database (CARD; version 3.2.8) [49]. The use of multiple tools and databases allows for cross-validation of the results, enhancing both accuracy and coverage in detecting known and novel ARGs across the genome.

#### 2.3.4. Pangenome Analysis

Pangenome analysis of the six isolates from this study was conducted using Roary version 3.13.0 [50] to determine the gene clusters and delineate the core and accessory genomes across the isolates. The analysis was executed using the command “roary -f roary_out -e -n -v -p 30 *.gff”, which enabled parallel processing, excluded splitting paralogs, and allowed for refined gene clustering. This approach facilitated the identification of shared and unique genetic elements among the isolates, providing insights into their genomic diversity and potential functional differences. Following the pangenome analysis, phylogenetic inference for both core and accessory genes was conducted using the maximum likelihood method implemented in IQ-TREE version 2.2.0 [51]. IQ-TREE analysis was performed with the command “iqtree2 -s core_gene_alignment.aln -m MFP -bb 1000 -alrt 1000 -nt 30”. This command employed the ModelFinder Plus algorithm for best-fit model selection, ensuring the most appropriate evolutionary model was used for the data. Additionally, 1000 ultrafast bootstrap replicates and SH-aLRT tests were conducted to assess branch support, providing robust statistical validation for the resulting phylogenetic trees.

## 3. Results

### 3.1. Data Summary

A comprehensive summary of the key metrics for each of the six mycobacterium isolates sequenced in this study is provided in Table 1. These metrics include the total number of clean reads, the sum of clean bases, the average read length, quality scores represented by Q20 and Q30, and GC content. The data reflect acceptable quality standards, and subsequent analyses were then conducted as described below.

### 3.2. Genome Assembly and Annotation

The results of de novo assembly and annotation are shown in Table 2, highlighting key genomic features, including genome size, the quality of assembled genomes, and the number of predicted genes. The analysis revealed high-quality draft genomes for all six isolates with genomic features that matched the respective reference genomes in terms of size and predicted gene count. However, the higher number of contigs observed in the annotated genomes is likely due to the limitations of short-read sequencing, which struggles to resolve repetitive regions and complex genomic structures, resulting in fragmented assemblies.

### 3.3. Species Identification

**Figure 1:**
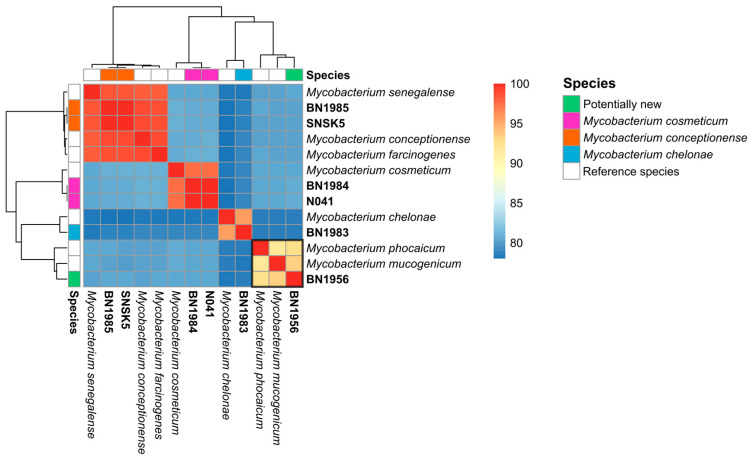
Genomic relatedness heat map. This figure displays the relationships between the study isolates and their tentative reference genomes based on 16S rRNA and MLST proposed taxa. The genomes were compared using ANI. A darker red color indicates higher similarity, while shades of blue suggest greater differences.

A comparison of species identification results based on the previous 16S rRNA sequence-based homology [11] and the current analysis using the assembled whole genome is summarized in Table 3. Initially, the following six isolates belonging to five different species were identified by DNA barcoding using the 16S rRNA amplicon sequence: *Mycobacterium chelonae* (BN1983), *M. cosmeticum* (BN1984 and N041), *M. farcinogenes* (SNSK5), *M. senegalense* (BN1985), and *M. mucogenicum* (BN1956). WGS and extended genomic comparisons with MLST, ANI, and dDDH led to the reclassification of these isolates (see Table 3). Isolates BN1985 and SNSK5 were identified as *M. conceptionense*, while BN1983 was confirmed as *M. chelonae* and BN1984 and N041 were confirmed as *M. cosmeticum*. There were discrepancies in species identification for some isolates when comparing the 16S rRNA sequences with the assembled draft genomes, particularly for isolates BN1985 and SNSK5. Analysis of isolate BN1956 revealed a previously unrecognized species that is proposed here as *M. mucogenicum* subsp. *phocaicum* sp. nov. The MLST analysis not only confirmed the identity of these isolates but also revealed similarities in the allele frequencies of housekeeping genes, particularly between isolates BN1984 and N041 and between isolates BN1985 and SNSK5. The similarity of the isolates was further supported by ANI (Figure 1) and dDDH scores (Supplementary Material Table S1). The MLST analysis also suggested that isolate BN1956 might represent a potential new species, as it had an ANI score < 95% compared to its closest relatives, implying that it is not the same species (Table 4).

### 3.4. Type-Based Species and Subspecies Clustering

**Figure 2:**
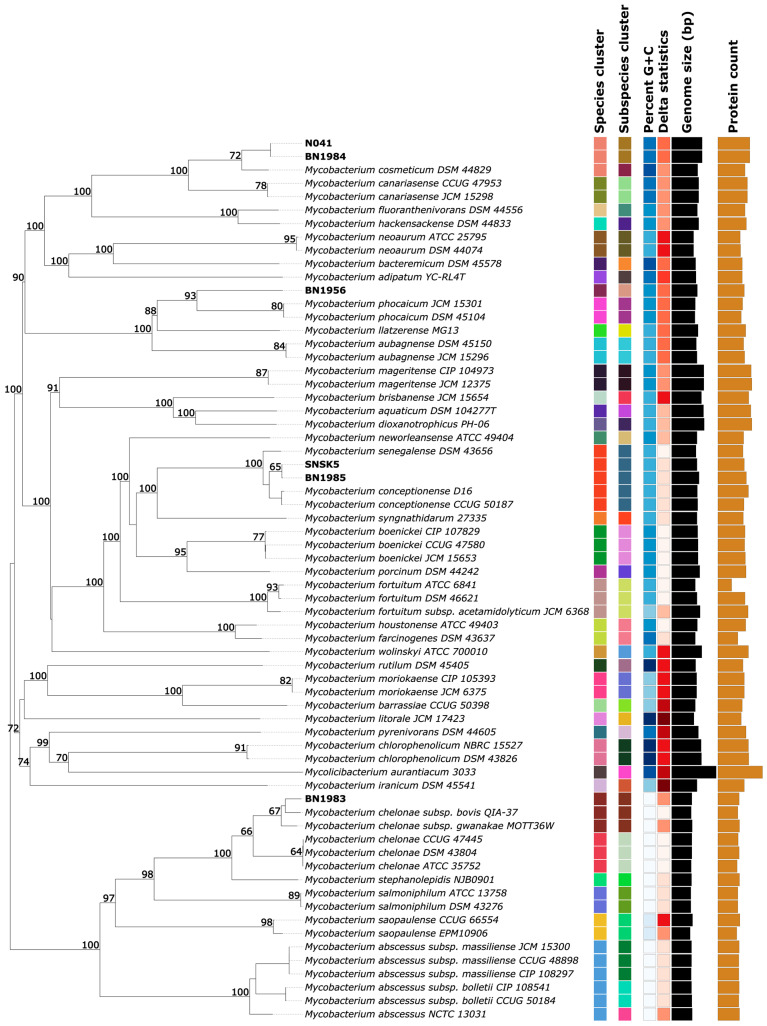
Tree derived with FastME 2.1.6.1 [34] from GBDP distances calculated from genome sequences. Branch lengths are scaled with respect to GBDP distance formula *d5*. The numbers above branches are GBDP pseudo-bootstrap support values > 60% from 100 replications, with an average branch support of 81.5%. The tree was rooted at the midpoint [35].

The taxonomic identification of the query strains, together with the respective results of dDDH, can be found in Supplementary Material Table S1. Isolates N041 and BN1984 formed a common cluster, indicating a close relationship, while isolates SNSK5 and BN1985 formed another distinct cluster (see Figure 2). Isolate BN1983 was clustered with its reference species, indicating a close evolutionary relationship. Notably, isolate BN1956 formed a separate cluster with a longer branch, suggesting a more distant relationship compared to the other isolates. This shows that our study species are included in 4 of the 40 subspecies clusters. Detailed information on the strains used for the phylogenetic analysis can be found in Supplementary Material Table S2.

### 3.5. Comparative Genomic Analysis

**Figure 3:**
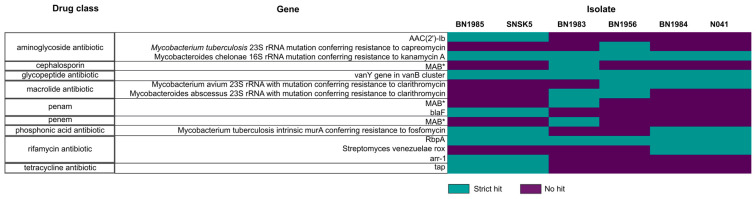
Distribution of antimicrobial resistance genes among the 6 *Mycobacterium* isolates in this study. Genes with an asterisk (*) appear more than once because they belong to more than one drug class in the Antibiotic Resistance Ontology (ARO). Strict hit represents genes with a bitscore ≥500 when matched to the reference database.

The analysis revealed plasmid sequences in the genomes of five isolates, except for isolate BN1983, and a BLAST search showed that these sequences matched significantly with those of other *Mycobacterium* species, suggesting a potential role for plasmid recombination within their natural context (Supplementary Material Table S3). Additionally, various ARGs were found in all six isolate genomes, conferring resistance to nine major drug classes (Figure 3). Furthermore, five major types of mobile genetic elements (MGEs) were detected in the genomes of the study isolates, including integration/excision elements, phages, replication/recombination/repair elements, transfer elements, and stability/transfer/defense elements. The MGEs were distributed across both putative plasmid and chromosome contigs. This highlights the importance of MGEs in horizontal gene transfer and the evolution of bacterial pathogens.

**Figure 4:**
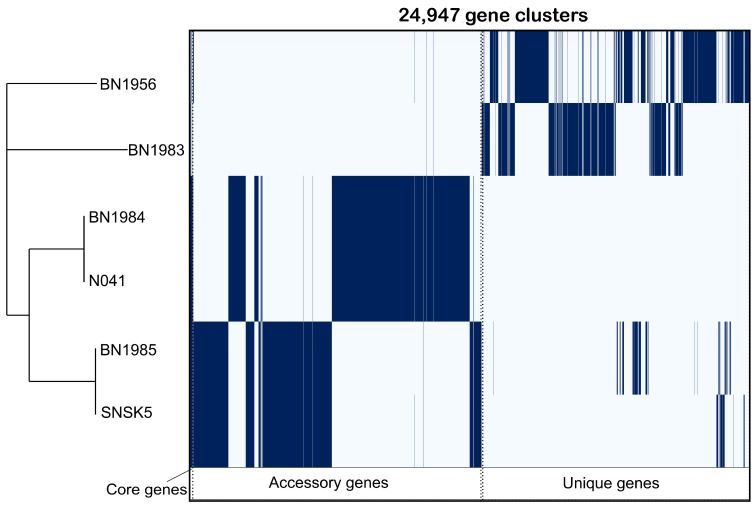
Pangenome analysis of *Mycobacterium* isolates. The analysis was based on six isolates, categorizing the genes into core genes (shared by all strains), accessory genes (present in some strains), and unique genes (specific to individual strains). Each blue bar represents a gene.

**Figure 5:**
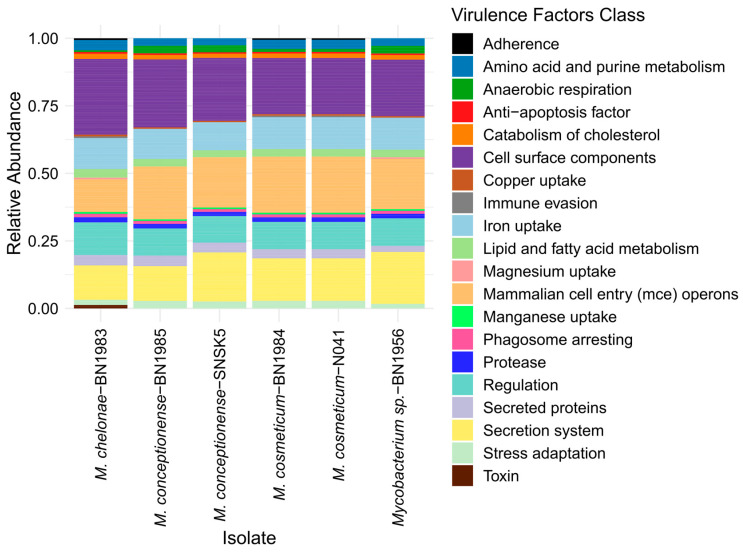
Illustration of the distribution of virulence factor classes among the six *Mycobacterium* isolates analyzed in this study. Each bar represents the relative abundance of the virulence factors and gives insight into the different virulence profiles of the isolates.

The pangenome analysis revealed a total of 24,947 gene clusters in the six isolates examined in this study. Among these, 25 were identified as core genes shared by all strains, while the remaining 24,922 were classified as shell genes (Figure 4). Remarkably, a considerable number of unique genes was detected in these isolates, underscoring the presence of isolate-specific genomic features. Functional annotation of the accessory genome revealed a variety of gene functions, including potential virulence factors, antibiotic resistance genes, and metabolic pathways. All six mycobacterial isolates in this study carried multiple virulence factors, as described in Supplementary Material Table S4. The distribution of these virulence factors varied between isolates, indicating differences in pathogenicity and adaptive mechanisms, as shown in Figure 5. The results indicated the presence of important virulence factors in *Mycobacterium*, such as the antigen 85 complex, *DevR*, and *DevS*, among many others. The findings were particularly intriguing, especially because *M. chelonae*-BN1983 exhibited virulence-associated toxin mycolactone, a feature that was absent in the other isolates. Additionally, *M. chelonae* BN1983 and *M. cosmeticum* (BN1984 and N041) were found to possess an additional class of adherence virulence factors encoded by the *groEL* gene, which distinguishes them from the other isolates analyzed in this study.

**Figure 6:**
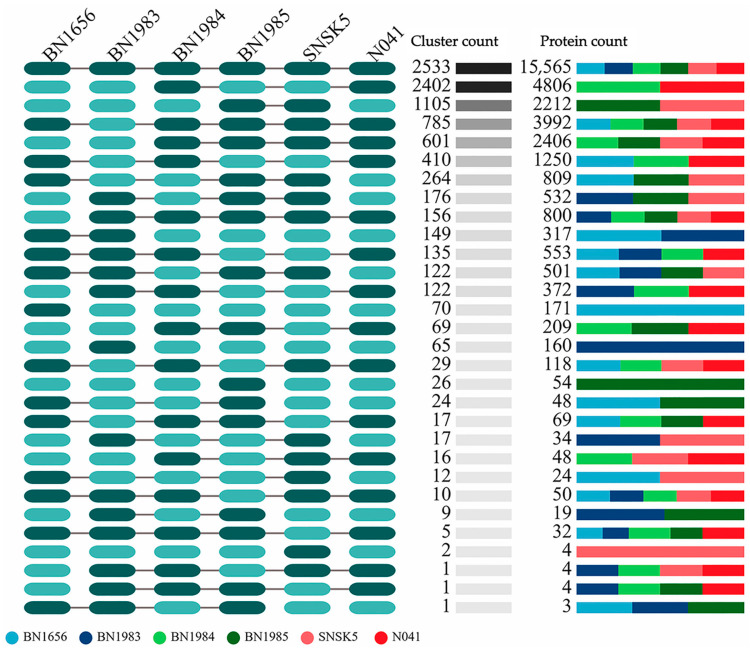
Illustration of the gene family clusters within the six mycobacterial isolates from this study. The diagram shows both the common and unique gene clusters with the respective number of proteins. The oval shapes in the inverted box on the left represent overlaps between the isolates. The dark-green shading represents overlapping gene families, while the light-green shading represents no overlap.

A comprehensive analysis of gene family clusters in six *Mycobacterium* isolates identified a total of 9334 clusters, of which 2533 occur together and 3215 occur individually (Figure 6). This analysis revealed different patterns in the number of unique gene clusters in the different species. Isolate BN1985, representing *M. conceptionense*, had 26 unique gene clusters, while another *M. conceptionense* isolate, SNSK5, had only 2 unique clusters. In contrast, *M. chelonae* isolate BN1983 had a significantly higher number of 65 unique gene clusters. Remarkably, *M. cosmeticum* isolates BN1984 and N041 had no unique gene clusters, indicating a greater similarity in gene content compared to other studied *Mycobacterium* species. The most significant discovery was a potential new *Mycobacterium* species represented by isolate BN1956, which had the highest number of unique gene clusters, at 70. These variations in the profiles of unique gene clusters illustrate the great genetic diversity within the *Mycobacterium* genus and provide insights into its evolutionary pathways and potential functional specializations.

## 4. Discussion

In this study, we present the complete genomic sequences of six mycobacterial isolates that were previously analyzed for their phenotypic properties and pathogenicity [11,14]. Our research highlights the limitations of the 16S rRNA gene in accurately distinguishing between closely related species. Whole genome sequencing overcomes this limitation by enabling a more detailed analysis. For instance, the use of whole genome ANI and phylogenetic analysis of core genes provided convincing evidence for the reclassification of isolate BN1956 as a potential new species. Although we have provisionally assigned the designation *Mycobacterium mucogenicum* subsp. *phocaicum* sp. nov. to isolate BN1956 based on comprehensive genomic analyses, formal recognition of this new species is contingent upon compliance with the *International Code of Nomenclature of Prokaryotes* (ICNP) [52]. This process involves the description of the species in the *International Journal of Systematic and Evolutionary Microbiology* (IJSEM), deposition of the species in international culture collections, and validation by *the International Committee on Systematics of Prokaryotes* (ICSP). The formal description of the species will be the focus of a subsequent investigation, requiring the completion of all prescribed taxonomic procedures. On the other hand, isolates BN1985 and SNSK5, which were initially identified by 16S rRNA analysis as *M. senegalense* and *M. farcinogenes*, respectively, were reclassified as *M. conceptionense* following whole genome sequencing. These results highlight the challenges and potential inaccuracies in identifying *Mycobacterium* species based solely on a single or limited number of target genes, emphasizing the importance of comprehensive genomic analysis for accurate taxonomic classification.

The accuracy of taxonomic classifications is fundamentally linked to the scope and quality of the reference databases used. This relationship is emphasized by the variability of taxonomic conclusions that can result from the use of different reference databases (see Table 3). To maintain consistency and ensure the reliability of our classifications, this study adhered to established standards for species delimitation by adopting ANI (average nucleotide identity) and dDDH (digital DNA–DNA hybridization) thresholds. Specifically, we followed a criterion of 95%+ coverage for ANI [19] and a threshold of 70% for dDDH [25], both of which are recognized as critical benchmarks for accurate species delimitation.

The identification of matching plasmid sequences in different *Mycobacterium* isolates in our study emphasizes the dynamic and adaptive nature of *Mycobacterium* genomes. It also underscores the crucial role of horizontal gene transfer in their evolutionary processes. This observation is supported by the findings of Chen et al. [53] and Redondo-Salvo et al. [54], which, together, emphasize the importance of plasmid-mediated genetic exchange for the rapid adaptation and diversification of bacterial species. All these results reflect the inherent genomic adaptability and shared evolutionary pathways between closely related bacterial species. Consequently, they prompt a re-evaluation of species delimitation criteria, taxonomic classification, and the influence of conserved plasmid sequences on functional traits. Furthermore, understanding the distribution of plasmids and their dynamic interactions is crucial for the assessment of potential risks associated with antibiotic resistance, virulence, and environmental adaptability [55]. Further research is essential to decipher the importance and functional impact of these plasmids within bacterial communities and their impact on ecological systems and public health. In addition, all six *Mycobacterium* isolates examined in this study exhibited a variety of ARGs that confer resistance to multiple classes of antibiotics. The results of in silico analysis are consistent with previously reported antibiotic susceptibility profiles for these isolates [11], highlighting the diversity of resistance mechanisms, including the alteration of antibiotic targets, the protection of these targets, inactivation of antibiotics, and the enhancement of antibiotic efflux. This multi-layered resistance underlines the complexity of dealing with these infections, requiring a deeper understanding of the underlying genetic mechanisms to develop effective treatment strategies.

The identification of virulence factors plays a central role in the assessment of bacterial pathogenicity, as these components are crucial for the ability of the bacterium to infect a host and cause clinical manifestation [56]. The genome sequence analysis of six isolates revealed the presence of significant number of virulence factors, including multiple copies of the antigen 85 complex (*Ag85*), which plays a key role in adherence. *Ag85*, which consists of mycolyltransferases found in mycobacteria, is instrumental in the pathogenesis of several species of this genus, particularly *Mycobacterium tuberculosis*, by contributing to the synthesis of the mycobacterial cell wall [57]. In addition, we identified the presence of *DevR* (also known as *DosR*) and *DevS*, regulatory proteins in mycobacteria that play a crucial role in the adaptation of the bacterium to environmental stress, especially under hypoxic conditions. The genes encoding proteins associated with “*mycobacterial*” virulence, such as the PE and PPE family proteins, MCE and YrbE proteins, were also detected in all six isolates, highlighting their widespread role in the virulence mechanism of these bacteria. These proteins play a crucial role in the adaptation strategy of the bacterium to survive under low-oxygen conditions [58]. All isolates possess catalase–peroxidase (*katG*), an enzyme of great importance for various mycobacterial species, including *M. tuberculosis*. *katG* is essential for the detoxification of reactive oxygen species and, thus, increases the bacterium’s resistance to oxidative stress [59]. The observed virulence factor profile is characteristic of the *Mycobacterium* genus and enables these bacteria to resist environmental stressors and effectively modulate or evade host immune defenses. *Mycobacterium chelonae* and *M. cosmeticum* appear to have acquired novel virulence genes from distantly related environmental bacteria, including *Klebsiella* sp., *Neisseria* sp., *Clostridium* sp., and *Streptococcus* sp. Horizontally transferred genes include the T6SS-II (*clpB*) system, as well as catalase (*katA*), GroEL (*groel*), and capsule (*rmlb*) genes. We hypothesize that these genes arose by horizontal gene transfer (HGT) and confer significant functional advantages to the recipient mycobacterial species. The T6SS-II system, for instance, is involved in bacterial pathogenesis and host–microbe interactions, potentially increasing the virulence of *M. chelonae* and *M. cosmeticum* [60]. Similarly, the catalase and GroEL genes may have enhanced the oxidative stress response and protein-folding ability of these bacteria, contributing to their environmental fitness [61].

These discoveries affirm the crucial role of HGT as an evolutionary mechanism that enables bacteria to rapidly acquire new genetic traits from diverse sources, influencing their phenotypic characteristics and ecological adaptations [62]. The identification of these genes in *M. chelonae* and *M. cosmeticum* underscores the importance of comparative genomics in unraveling the complex evolutionary pathways of non-tuberculous mycobacterial species. Notably, *M. chelonae*-BN1983 was the only isolate in our study that produced mycolactones, which are complex macrolide toxins typically associated with *Mycobacterium ulcerans* and linked to *Buruli ulcer*, a necrotizing skin disease. These toxins have immunosuppressive and cytotoxic effects [63]. The elevated mortality rate observed in *B. splendens* following experimental exposure to *M. chelonae*-BN1983 [11,12] could be attributed to the strain’s unique genetic profile, which includes mycolactone-encoding genes responsible for producing potent toxins usually found in highly virulent mycobacterial species. Furthermore, its genome contains numerous genes encoding cell surface components, including members of the Mycobacterial membrane protein Large (*MmpL*) family, such as *MmpL4a*. *MmpL* proteins, particularly *MmpL4a*, which are known to play key roles in mycobacterial virulence and drug resistance [64], suggesting that this strain possesses a unique potential for drug resistance and high virulence.

## 5. Conclusions

In summary, this study provides valuable insights into the genomic characterization of six rapidly growing non-tuberculous mycobacteria (RGM) from Siamese fighting fish (*Betta splendens*). Initial identification via 16S rRNA sequences revealed taxonomic ambiguities that led to comprehensive whole genome sequencing (WGS) and advanced genomic analyses, such as multilocus sequence typing (MLST), average nucleotide identity (ANI), and digital DNA–DNA hybridization (dDDH). These methods led to the reclassification of certain species and the identification of a potential new species, namely *Mycobacterium mucogenicum* subsp. *phocaicum* sp. nov. The detection of shared virulence genes and horizontally acquired virulence factors in *M. chelonae* and *M. cosmeticum* emphasizes the pathogenicity and adaptability of these bacteria. This study provides the first draft genome sequences of RGM isolates in *B. splendens* and highlights the crucial role of WGS in understanding the genetic diversity and pathogenic potential of RGMs, contributing to improved diagnostic, therapeutic, and health management strategies for these infections.

## Figures and Tables

**Table 1:** Quality statistics of sequence data generated in this study.

Sample ID	Clean Reads	Clean Bases (bp)	Read Length	Q20 (%)	Q30 (%)	GC (%)
BN1956	4,050,677	1,215,203,100	PE150	95.00	87.02	66.64
BN1983	4,043,521	1,213,056,300	PE150	95.42	87.77	64.06
BN1984	4,037,827	1,211,348,100	PE150	95.09	87.31	64.05
BN1985	4,038,265	1,211,479,500	PE150	95.16	87.36	65.94
SNSK5	4,037,574	1,211,272,200	PE150	95.22	87.73	66.22
N041	4,048,072	1,214,421,621	PE150	95.22	87.69	67.27

**Table 2:** Genomic feature distribution of the six bacterial isolates in this study.

Features		Isolates					
		BN1956	BN1983	BN1984	BN1985	SNSK5	N041
GenBank accession		JAYXBQ000000000	JAYXBR000000000	JAYXBS000000000	JAYXBT000000000	JAYXBU000000000	JAYXBV000000000
Size (bp)		6,369,566	5,056,078	7,557,134	6,818,352	6,342,755	7,556,912
Minimum sequence length		350	374	361	378	378	361
Maximum sequence length		446,856	1,063,941	1,115,510	1,179,774	903,710	861,703
Contigs		84	19	57	39	30	59
N50 (bp)		205,143	800,484	398,421	540,107	520,742	385,935
Completeness (CheckM) (%)		99.32	99.48	99.62	100	99.66	99.62
Contamination (%)		1.06	0.38	5.29	1.15	0.98	5.29
L50		10	2	7	5	5	8
Genes		6184	4937	7512	6644	6118	7511
CDSs		6088	4885	7425	6558	6063	7424
Genes (coding)		6010	4854	7324	6506	6013	7322
Genes (RNA)		96	52	87	86	55	87
rRNA	*5S*	2	1	2	2	2	2
	*16S*	2	1	2	1	1	2
	*23S*	6	1	3	2	2	3
tRNAs		83	46	77	77	47	77
ncRNAs		3	3	3	4	3	3
Pseudo Genes		78	31	101	52	50	102

**Table 3:** Bacterial species identification based on 16S rRNA sequence homology compared to the assembled whole genome analyzed using multiple pipelines and databases.

Sample ID	16s rRNA *	MLST	PubMLST	TYGS	RAPT	DFAST	Conclusion
BN1956	*M. mucogenicum*	Mycobacteria	*M. phocaicum*	Potential new species	Inconclusive	Inconclusive	*M. mucogenicum* subsp. *phocaicum* sp. nov.
BN1983	*M. chelonae*	Mycobacteria	*M. chelonae*	*M. chelonae*	*M. chelonae*	*M. chelonae*	*M. chelonae*
BN1984	*M. cosmeticum*	Mycobacteria	*M. cosmeticum*	*M. cosmeticum*	*M. cosmeticum*	*M. cosmeticum*	*M. cosmeticum*
BN1985	*M. senegalense*	Mycobacteria	*M. conceptionense*	*M. senegalense*	*M. conceptionense*	*M. conceptionense*	*M. conceptionense*
SNSK5	*M. farcinogenes*	Mycobacteria	*M. conceptionense*	*M. senegalense*	*M. conceptionense*	*M. conceptionense*	*M. conceptionense*
N041	*M. cosmeticum*	Mycobacteria	*M. cosmeticum*	*M. cosmeticum*	*M. cosmeticum*	*M. cosmeticum*	*M. cosmeticum*

* based on the work of Dinh-Hung et al. [11].

**Table 4:** ANI scores of isolate BN1956 against two closely related reference strains.

Isolates	BN1956	*Mycobacterium mucogenicum*	*Mycobacterium phocaicum*
GenBank accession	JAYXBQ000000000	GCF_005670685.2	GCF_010731115.1
Size (bp)	6,326,040	6,098,580	5,853,197
GC (%)	67.11	67.23	67.05
OrthoANIu (%)		93.26	92.51
dDDH (d4, in %)		51.0	70.3

## Data Availability

The data that support the findings of this study are available upon request. The draft genomes of the six isolates were submitted to NCBI under BioProject PRJNA1063977.

## Supplementary Materials

The following supporting information can be downloaded at: https://www.mdpi.com/article/10.3390/ani14192833/s1, Table S1: Pairwise comparison for dDDH; Table S2: Strains for phylogeny; Table S3: Plasmids; Table S4: Virulence factors.
